# *trans*-Diaqua­bis[5-carb­oxy-4-carboxyl­ato-2-(4-pyridinio)-1*H*-imidazol-1-ido-κ^2^ *N* ^3^,*O* ^4^]iron(II)

**DOI:** 10.1107/S160053680902337X

**Published:** 2009-06-24

**Authors:** Xia Li, Wei Liu, Ben-Lai Wu, Hong-Yun Zhang

**Affiliations:** a Department of Chemistry and Chemical Engineering, Pingdingshan Engineering College, Pingdingshan, Henan, 467001, People’s Republic of China; b Department of Chemistry, Zhengzhou University, Zhengzhou, Henan, 450052, People’s Republic of China

## Abstract

In the title complex, [Fe(C_10_H_6_N_3_O_4_)_2_(H_2_O)_2_], the Fe^II^ atom is located on a twofold rotation axis and is coordinated by two *trans*-positioned *N*,*O*-bidentate and zwitterionic 5-carboxy-2-(pyridinium-4-yl)-1*H*-imidazol-1-ide-4-carboxylate H_2_PIDC^−^ ligands and two water mol­ecules in a distorted environment. In the crystal packing, a three-dimensional network is constructed *via* hydrogen-bonding involving the water mol­ecules, uncoordinated imidazole N atom, protonated pyridine N and carboxyl­ate O atoms.

## Related literature

For the use of the multifunctional connector 4,5-imidazole­dicarboxylic acid (H_3_IDC) in coordination chemistry, see: Liu *et al.* (2004 ▶); Maji *et al.* (2005 ▶); Plieger *et al.* (2005 ▶); Rajendiran *et al.* (2003 ▶); Zou *et al.* (2005 ▶). For the preparation of 2-(pyridin-4-yl)-1*H*-imidazole-4,5-dicarboxylic acid, see: Sun *et al.* (2006 ▶).
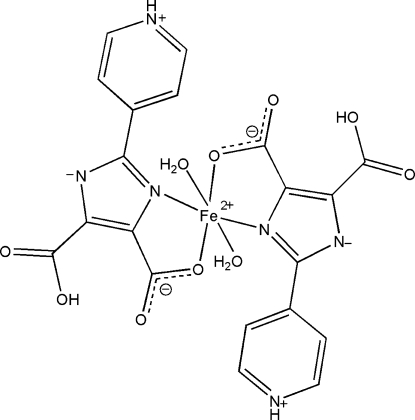


## Experimental

### 

#### Crystal data

[Fe(C_10_H_6_N_3_O_4_)_2_(H_2_O)_2_]
*M* *_r_* = 556.24
Monoclinic, 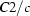

*a* = 21.344 (4) Å
*b* = 7.3900 (15) Å
*c* = 13.768 (3) Å
β = 104.70 (3)°
*V* = 2100.6 (7) Å^3^
*Z* = 4
Mo *K*α radiation
μ = 0.80 mm^−1^
*T* = 173 K
0.25 × 0.15 × 0.12 mm

#### Data collection

Rigaku Mercury CCD diffractometer
Absorption correction: multi-scan (*CrystalClear*; Rigaku, 2000 ▶) *T*_min_ = 0.870, *T*_max_ = 0.921
8952 measured reflections
2386 independent reflections
2083 reflections with *I* > 2σ(*I*)
*R*_int_ = 0.021

#### Refinement

*R*[*F* ^2^ > 2σ(*F* ^2^)] = 0.029
*wR*(*F* ^2^) = 0.079
*S* = 1.04
2386 reflections
175 parameters
H atoms treated by a mixture of independent and constrained refinement
Δρ_max_ = 0.37 e Å^−3^
Δρ_min_ = −0.25 e Å^−3^

### 

Data collection: *CrystalClear* (Rigaku, 2000 ▶); cell refinement: *CrystalClear*; data reduction: *CrystalClear*; program(s) used to solve structure: *SHELXS97* (Sheldrick, 2008 ▶); program(s) used to refine structure: *SHELXL97* (Sheldrick, 2008 ▶); molecular graphics: *SHELXTL* (Sheldrick, 2008 ▶); software used to prepare material for publication: *SHELXTL*.

## Supplementary Material

Crystal structure: contains datablocks I, global. DOI: 10.1107/S160053680902337X/kp2221sup1.cif

Structure factors: contains datablocks I. DOI: 10.1107/S160053680902337X/kp2221Isup2.hkl

Additional supplementary materials:  crystallographic information; 3D view; checkCIF report

## Figures and Tables

**Table 1:** Selected bond lengths (Å)

Fe1—O6	2.081 (2)
Fe1—O1	2.1087 (13)
Fe1—O7	2.121 (2)
Fe1—N1	2.2311 (14)

**Table 2:** Hydrogen-bond geometry (Å, °)

*D*—H⋯*A*	*D*—H	H⋯*A*	*D*⋯*A*	*D*—H⋯*A*
O6—H6⋯N2^i^	0.86 (2)	2.04 (2)	2.8806 (18)	169 (3)
N3—H3⋯O2^ii^	0.88	1.99	2.707 (2)	138
O3—H3*B*⋯O2	0.99	1.53	2.4959 (19)	166

Symmetry codes: (i) 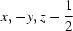; (ii) 
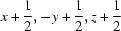
.

**Fig. 1.**
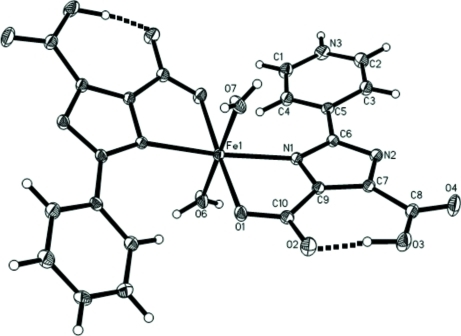
A view of the complex of (I) showing the atom-labelling scheme and displacement ellipsoids at the 30% probability level.

**Fig. 2.**
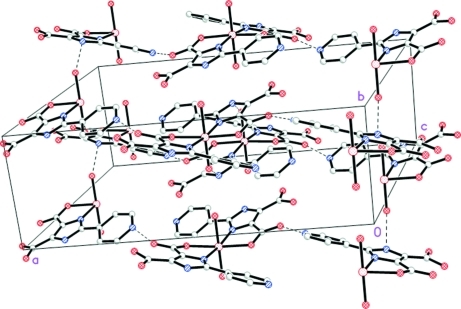
The crystal packing of (I) showing the three-dimensional hydrogen-bonding network; H atoms not involved in hydrogen bonding have been omited.

## supplementary crystallographic information

### Comment

Multifunctional connector 4,5-imidazoledicarboxylic acid (H_3_IDC), adjusting its existing forms and coordination modes through pH or temperature in assembly reaction systems, shows more interesting traits in the construction of nano-structures and MOFs, and thus has been extensively investigated in coordination chemistry (Maji *et al.*, 2005; Liu *et al.*, 2004; Zou *et al.*, 2005; Rajendiran *et al.*, 2003; Plieger *et al.*, 2005). 2-(Pyridin-4-yl)-1*H*-imidazole-4,5-dicarboxylate acid (H_3_PIDC), a close analogue of H_3_IDC, is endowed a promising building block H_3_IDC and the additional pyridine group modulate coordination ability to give more potential coordination modes and enlarge conjugation system. In order to explore the coordination chemistry of this ligand, we have isolated a new Fe^II^ complex, [Fe(H_2_PIDC)_2_(H_2_O)_2_], (I), by the reaction of H_3_PIDC and Fe^II^ sulfate under the hydrothermal condition. We report here the single-crystal structure of this complex.

The molecule of (I) is a discrete neutral monomer  (Fig. 1) in which the Fe atom resides on a twofold rotation axes  and the asymmetric unit comprises  a half of the [Fe(H_2_PIDC)_2_(H_2_O)_2_] formula unit. Each Fe atom is hexacoordinated by N_2_O_4_ with two chelating rings from two H_2_PIDC ligands arranged symmetrically in the equatorial plane and two water molecules occupying the apical sites, defining an octahedral coordination (Table 1). In this complex, a carboxyl group and imidazole group are deprotonated and the pyridyl group is protonated, and the ligand bears a formal charge of -1. The free carboxylate atoms O3 and O2 form an intramolecular hydrogen bond (Table 2). All non-H atoms in the imidazole-4,5-dicarboxyl group are nearly coplanar [the mean deviation is 0.025 (4) Å], and the dihedral angle between imidazole group and pyridine group is 10.3 (2)°.

The hydrogen bonding involves the water molecules, uncoordinated imidazole N atom, protonated pyridine N and carboxylate O atoms (Table 2 and Fig. 2).

### Experimental

A mixture of Fe^II^  sulfate (0.028 g, 0.1 mmol), 2-(pyridin-4-yl)-1*H*-imidazole-4,5-dicarboxylic acid (0.024 g, 0.1 mmol) (Sun *et al.*, 2006), NaOH (0.004 g, 0.1 mmol) and H2O (10 ml) was sealed into a Teflon-lined stainless autoclave and heated at 423 K for 3 d, then cooled to room temperature gradually and red block crystals of (I) were obtained.

### Refinement

H atoms attached to N and O atoms were located in a difference Fourier maps and refined as riding in their as-found relative positions, with *U*_iso_(H) = 1.5*U*_eq_(O,*N*). Other H atoms were positioned geometrically with C—H = 0.95 Å and constrained to ride on their parent atoms with *U*_iso_(H) = 1.2*U*_eq_(C).

### Crystal data

**Table:**

[Fe(C_10_H_6_N_3_O_4_)_2_(H_2_O)_2_]	*F*(000) = 1136
*M**_r_* = 556.24	*D*_x_ = 1.759 Mg m^−^^3^
Monoclinic, *C*2/*c*	Mo *K*α radiation, λ = 0.71073 Å
*a* = 21.344 (4) Å	θ = 2.9–28.3°
*b* = 7.3900 (15) Å	µ = 0.80 mm^−^^1^
*c* = 13.768 (3) Å	*T* = 173 K
β = 104.70 (3)°	Block, red
*V* = 2100.6 (7) Å^3^	0.25 × 0.15 × 0.12 mm
*Z* = 4	

### Data collection

**Table:**

Mercury CCD diffractometer	2386 independent reflections
Radiation source: fine-focus sealed tube	2083 reflections with *I* > 2σ(*I*)
graphite	*R*_int_ = 0.021
ω scans	θ_max_ = 27.5°, θ_min_ = 2.9°
Absorption correction: multi-scan (*CrystalClear*; Rigaku, 2000)	*h* = −27→27
*T*_min_ = 0.870, *T*_max_ = 0.921	*k* = −9→9
8952 measured reflections	*l* = −17→17

### Refinement

**Table:**

Refinement on *F*^2^	Primary atom site location: structure-invariant direct methods
Least-squares matrix: full	Secondary atom site location: difference Fourier map
*R*[*F*^2^ > 2σ(*F*^2^)] = 0.029	Hydrogen site location: inferred from neighbouring sites
*wR*(*F*^2^) = 0.079	H atoms treated by a mixture of independent and constrained refinement
*S* = 1.04	*w* = 1/[σ^2^(*F*_o_^2^) + (0.0388*P*)^2^ + 2.1264*P*] where *P* = (*F*_o_^2^ + 2*F*_c_^2^)/3
2386 reflections	(Δ/σ)_max_ < 0.001
175 parameters	Δρ_max_ = 0.37 e Å^−^^3^
0 restraints	Δρ_min_ = −0.25 e Å^−^^3^

### Special details

**Table:**

Geometry. All e.s.d.'s (except the e.s.d. in the dihedral angle between two l.s. planes) are estimated using the full covariance matrix. The cell e.s.d.'s are taken into account individually in the estimation of e.s.d.'s in distances, angles and torsion angles; correlations between e.s.d.'s in cell parameters are only used when they are defined by crystal symmetry. An approximate (isotropic) treatment of cell e.s.d.'s is used for estimating e.s.d.'s involving l.s. planes.
Refinement. Refinement of *F*^2^ against ALL reflections. The weighted *R*-factor *wR* and goodness of fit *S* are based on *F*^2^, conventional *R*-factors *R* are based on *F*, with *F* set to zero for negative *F*^2^. The threshold expression of *F*^2^ > σ(*F*^2^) is used only for calculating *R*-factors(gt) *etc*. and is not relevant to the choice of reflections for refinement. *R*-factors based on *F*^2^ are statistically about twice as large as those based on *F*, and *R*- factors based on ALL data will be even larger.

### Fractional atomic coordinates and isotropic or equivalent isotropic displacement parameters (Å^2^)

**Table:**

	*x*	*y*	*z*	*U*_iso_*/*U*_eq_	
Fe1	0.0000	0.19391 (5)	0.2500	0.02067 (11)	
O1	−0.10126 (6)	0.20613 (19)	0.22910 (9)	0.0309 (3)	
O2	−0.17764 (6)	0.3056 (2)	0.29923 (10)	0.0391 (4)	
O3	−0.18924 (6)	0.3890 (2)	0.46949 (10)	0.0406 (4)	
H3B	−0.1867	0.3754	0.3994	0.061*	
O4	−0.12932 (7)	0.3875 (2)	0.62503 (10)	0.0452 (4)	
O6	0.0000	−0.0877 (3)	0.2500	0.0352 (5)	
N1	−0.00912 (6)	0.21688 (19)	0.40741 (10)	0.0203 (3)	
N2	−0.01854 (7)	0.2842 (2)	0.56415 (10)	0.0236 (3)	
N3	0.21933 (7)	0.1542 (2)	0.63905 (12)	0.0341 (4)	
H3	0.2608	0.1397	0.6677	0.051*	
C1	0.19580 (9)	0.1005 (3)	0.54346 (14)	0.0334 (4)	
H1	0.2237	0.0464	0.5079	0.040*	
C2	0.18128 (9)	0.2296 (3)	0.69186 (14)	0.0360 (5)	
H2	0.1994	0.2653	0.7595	0.043*	
C3	0.11686 (9)	0.2556 (3)	0.64969 (13)	0.0308 (4)	
H3A	0.0903	0.3092	0.6877	0.037*	
C4	0.13117 (8)	0.1242 (3)	0.49733 (13)	0.0281 (4)	
H4	0.1144	0.0871	0.4296	0.034*	
C5	0.09009 (8)	0.2030 (2)	0.55010 (12)	0.0220 (3)	
C6	0.02077 (8)	0.2333 (2)	0.50603 (12)	0.0210 (3)	
C7	−0.07755 (8)	0.3048 (2)	0.49880 (12)	0.0215 (3)	
C8	−0.13379 (8)	0.3626 (3)	0.53665 (13)	0.0272 (4)	
C9	−0.07183 (7)	0.2631 (2)	0.40239 (12)	0.0205 (3)	
C10	−0.11994 (8)	0.2578 (2)	0.30365 (12)	0.0244 (4)	
O7	0.0000	0.4810 (3)	0.2500	0.0340 (4)	
H6	−0.0101 (12)	−0.153 (3)	0.1969 (18)	0.051*	
H7	0.0223 (11)	0.530 (4)	0.2972 (17)	0.051*	

### Atomic displacement parameters (Å^2^)

**Table:**

	*U*^11^	*U*^22^	*U*^33^	*U*^12^	*U*^13^	*U*^23^
Fe1	0.01809 (17)	0.0276 (2)	0.01644 (17)	0.000	0.00460 (12)	0.000
O1	0.0218 (6)	0.0513 (8)	0.0176 (6)	−0.0004 (6)	0.0017 (5)	−0.0067 (5)
O2	0.0155 (6)	0.0730 (11)	0.0251 (7)	0.0076 (6)	−0.0012 (5)	−0.0053 (6)
O3	0.0198 (6)	0.0712 (10)	0.0312 (7)	0.0084 (6)	0.0071 (5)	−0.0069 (7)
O4	0.0391 (8)	0.0752 (11)	0.0249 (7)	0.0073 (8)	0.0144 (6)	−0.0062 (7)
O6	0.0571 (13)	0.0273 (10)	0.0212 (9)	0.000	0.0096 (9)	0.000
N1	0.0154 (6)	0.0279 (7)	0.0162 (6)	0.0011 (5)	0.0016 (5)	0.0006 (5)
N2	0.0188 (7)	0.0338 (8)	0.0170 (7)	−0.0013 (6)	0.0023 (5)	0.0005 (6)
N3	0.0156 (7)	0.0524 (10)	0.0296 (8)	0.0029 (7)	−0.0026 (6)	0.0055 (7)
C1	0.0236 (9)	0.0457 (11)	0.0315 (10)	0.0055 (8)	0.0079 (7)	0.0021 (8)
C2	0.0238 (9)	0.0558 (13)	0.0231 (9)	−0.0022 (8)	−0.0038 (7)	−0.0021 (8)
C3	0.0217 (9)	0.0473 (11)	0.0215 (9)	−0.0002 (8)	0.0022 (7)	−0.0035 (8)
C4	0.0224 (8)	0.0391 (10)	0.0212 (8)	0.0019 (7)	0.0025 (7)	0.0008 (7)
C5	0.0173 (8)	0.0277 (9)	0.0190 (8)	−0.0009 (6)	0.0013 (6)	0.0039 (6)
C6	0.0179 (8)	0.0274 (8)	0.0161 (7)	−0.0003 (6)	0.0015 (6)	0.0017 (6)
C7	0.0179 (7)	0.0285 (9)	0.0177 (7)	−0.0015 (6)	0.0035 (6)	−0.0004 (6)
C8	0.0230 (8)	0.0360 (10)	0.0235 (8)	−0.0004 (7)	0.0080 (7)	−0.0019 (7)
C9	0.0151 (7)	0.0280 (8)	0.0174 (8)	0.0003 (6)	0.0020 (6)	0.0005 (6)
C10	0.0173 (8)	0.0347 (9)	0.0189 (8)	−0.0013 (7)	0.0004 (6)	−0.0005 (7)
O7	0.0447 (12)	0.0303 (10)	0.0206 (9)	0.000	−0.0033 (8)	0.000

### Geometric parameters (Å, °)

**Table:**

Fe1—O6	2.081 (2)	N3—C2	1.341 (3)
Fe1—O1	2.1087 (13)	N3—C1	1.344 (2)
Fe1—O1^i^	2.1087 (13)	N3—H3	0.8793
Fe1—O7	2.121 (2)	C1—C4	1.376 (2)
Fe1—N1	2.2311 (14)	C1—H1	0.9500
Fe1—N1^i^	2.2311 (14)	C2—C3	1.364 (3)
O1—C10	1.251 (2)	C2—H2	0.9500
O2—C10	1.268 (2)	C3—C5	1.400 (2)
O3—C8	1.318 (2)	C3—H3A	0.9500
O3—H3B	0.9857	C4—C5	1.399 (2)
O4—C8	1.210 (2)	C4—H4	0.9500
O6—H6	0.86 (2)	C5—C6	1.467 (2)
N1—C6	1.351 (2)	C7—C9	1.398 (2)
N1—C9	1.366 (2)	C7—C8	1.488 (2)
N2—C6	1.352 (2)	C9—C10	1.482 (2)
N2—C7	1.358 (2)	O7—H7	0.79 (2)
			
O6—Fe1—O1	92.45 (4)	N3—C2—C3	120.77 (17)
O6—Fe1—O1^i^	92.45 (4)	N3—C2—H2	119.6
O1—Fe1—O1^i^	175.09 (8)	C3—C2—H2	119.6
O6—Fe1—O7	180.0	C2—C3—C5	119.65 (18)
O1—Fe1—O7	87.55 (4)	C2—C3—H3A	120.2
O1^i^—Fe1—O7	87.55 (4)	C5—C3—H3A	120.2
O6—Fe1—N1	94.36 (4)	C1—C4—C5	120.01 (16)
O1—Fe1—N1	77.89 (5)	C1—C4—H4	120.0
O1^i^—Fe1—N1	101.73 (6)	C5—C4—H4	120.0
O7—Fe1—N1	85.64 (4)	C4—C5—C3	118.05 (15)
O6—Fe1—N1^i^	94.36 (4)	C4—C5—C6	123.22 (15)
O1—Fe1—N1^i^	101.73 (6)	C3—C5—C6	118.73 (15)
O1^i^—Fe1—N1^i^	77.89 (5)	N1—C6—N2	114.46 (14)
O7—Fe1—N1^i^	85.64 (4)	N1—C6—C5	124.85 (15)
N1—Fe1—N1^i^	171.27 (7)	N2—C6—C5	120.68 (14)
C10—O1—Fe1	115.47 (11)	N2—C7—C9	108.34 (14)
C8—O3—H3B	114.1	N2—C7—C8	119.69 (14)
Fe1—O6—H6	124.1 (17)	C9—C7—C8	131.97 (15)
C6—N1—C9	103.60 (13)	O4—C8—O3	120.70 (17)
C6—N1—Fe1	147.96 (11)	O4—C8—C7	122.08 (16)
C9—N1—Fe1	107.20 (10)	O3—C8—C7	117.21 (15)
C6—N2—C7	104.46 (13)	N1—C9—C7	109.12 (14)
C2—N3—C1	121.75 (16)	N1—C9—C10	118.80 (14)
C2—N3—H3	119.1	C7—C9—C10	132.06 (15)
C1—N3—H3	119.2	O1—C10—O2	123.61 (15)
N3—C1—C4	119.76 (17)	O1—C10—C9	118.02 (15)
N3—C1—H1	120.1	O2—C10—C9	118.37 (15)
C4—C1—H1	120.1	Fe1—O7—H7	117.2 (19)
			
O6—Fe1—O1—C10	108.54 (13)	C7—N2—C6—N1	−0.9 (2)
O1^i^—Fe1—O1—C10	−71.46 (13)	C7—N2—C6—C5	178.14 (15)
O7—Fe1—O1—C10	−71.46 (13)	C4—C5—C6—N1	−11.1 (3)
N1—Fe1—O1—C10	14.60 (13)	C3—C5—C6—N1	168.70 (17)
N1^i^—Fe1—O1—C10	−156.48 (13)	C4—C5—C6—N2	169.95 (17)
O6—Fe1—N1—C6	92.7 (2)	C3—C5—C6—N2	−10.3 (3)
O1—Fe1—N1—C6	−175.7 (2)	C6—N2—C7—C9	0.63 (19)
O1^i^—Fe1—N1—C6	−0.7 (2)	C6—N2—C7—C8	−179.49 (16)
O7—Fe1—N1—C6	−87.3 (2)	N2—C7—C8—O4	−2.2 (3)
N1^i^—Fe1—N1—C6	−87.3 (2)	C9—C7—C8—O4	177.7 (2)
O6—Fe1—N1—C9	−103.82 (10)	N2—C7—C8—O3	176.82 (16)
O1—Fe1—N1—C9	−12.24 (11)	C9—C7—C8—O3	−3.3 (3)
O1^i^—Fe1—N1—C9	162.76 (11)	C6—N1—C9—C7	−0.38 (18)
O7—Fe1—N1—C9	76.18 (10)	Fe1—N1—C9—C7	−171.43 (11)
N1^i^—Fe1—N1—C9	76.18 (10)	C6—N1—C9—C10	−179.12 (15)
C2—N3—C1—C4	0.6 (3)	Fe1—N1—C9—C10	9.83 (18)
C1—N3—C2—C3	−0.4 (3)	N2—C7—C9—N1	−0.2 (2)
N3—C2—C3—C5	0.0 (3)	C8—C7—C9—N1	179.98 (17)
N3—C1—C4—C5	−0.4 (3)	N2—C7—C9—C10	178.35 (17)
C1—C4—C5—C3	0.0 (3)	C8—C7—C9—C10	−1.5 (3)
C1—C4—C5—C6	179.77 (17)	Fe1—O1—C10—O2	166.15 (15)
C2—C3—C5—C4	0.2 (3)	Fe1—O1—C10—C9	−13.9 (2)
C2—C3—C5—C6	−179.60 (18)	N1—C9—C10—O1	1.9 (2)
C9—N1—C6—N2	0.84 (19)	C7—C9—C10—O1	−176.46 (18)
Fe1—N1—C6—N2	164.57 (16)	N1—C9—C10—O2	−178.12 (16)
C9—N1—C6—C5	−178.20 (16)	C7—C9—C10—O2	3.5 (3)
Fe1—N1—C6—C5	−14.5 (3)		

Symmetry codes: (i) −*x*, *y*, −*z*+1/2.

### Hydrogen-bond geometry (Å, °)

**Table:**

*D*—H···*A*	*D*—H	H···*A*	*D*···*A*	*D*—H···*A*
O6—H6···N2^ii^	0.86 (2)	2.04 (2)	2.8806 (18)	169 (3)
N3—H3···O2^iii^	0.88	1.99	2.707 (2)	138
O3—H3B···O2	0.99	1.53	2.4959 (19)	166

Symmetry codes: (ii) *x*, −*y*, *z*−1/2; (iii) *x*+1/2, −*y*+1/2, *z*+1/2.
